# Clinical, Pathological and Molecular Insights on KRAS, NRAS, BRAF, PIK3CA and TP53 Mutations in Metastatic Colorectal Cancer Patients from Northeastern Romania

**DOI:** 10.3390/ijms241612679

**Published:** 2023-08-11

**Authors:** Vlad-Adrian Afrăsânie, Mihai-Vasile Marinca, Bogdan Gafton, Teodora Alexa-Stratulat, Alexandra Rusu, Eliza-Maria Froicu, Daniel Sur, Cristian Virgil Lungulescu, Larisa Popovici, Andrei-Vlad Lefter, Irina Afrăsânie, Anca-Viorica Ivanov, Lucian Miron, Cristina Rusu

**Affiliations:** 1 Department of Medical Oncology, Regional Institute of Oncology, 700483 Iasi, Romaniarusu.alexandra@email.umfiasi.ro (A.R.); andrei13vlad@yahoo.com (A.-V.L.); lucmir@gmail.com (L.M.); 2 Department of Oncology, Faculty of Medicine, “Grigore T. Popa” University of Medicine and Pharmacy, 700115 Iasi, Romania; 3 Department of Medical Oncology, The Oncology Institute “Prof. Dr. Ion Chiricuta”, 400015 Cluj-Napoca, Romania; dr.geni@yahoo.co.uk; 4 11th Department of Medical Oncology, “Iuliu Hatieganu” University of Medicine and Pharmacy, 400347 Cluj-Napoca, Romania; 5 Department of Oncology, University of Medicine and Pharmacy of Craiova, 200349 Craiova, Romania; 6 Department of Cardiology, Emergency Clinical Hospital “Sf. Spiridon”, 700111 Iasi, Romania; irina-demsa@email.umfiasi.ro; 7 Department of Pediatrics, Faculty of Medicine, “Grigore T. Popa” University of Medicine and Pharmacy, 700115 Iasi, Romania; anca_vi@yahoo.com; 8 Department of Genetics, Faculty of Medicine, “Grigore T. Popa” University of Medicine and Pharmacy, 700115 Iasi, Romania

**Keywords:** metastatic colorectal cancer, RAS, BRAF, PIK3CA, TP53

## Abstract

Mutations in RAS, BRAF, PIK3CA, and TP53 are well-established genetic abnormalities in metastatic colorectal cancer (mCRC). However, limited information is available for patients from Eastern Europe, including Romania. In this retrospective analysis, we investigated 104 mCRC patients from the Northeastern region of Romania to determine the frequency, distribution, coexistence, and clinicopathological and molecular correlations of these mutations. TP53 was the most frequently mutated gene (73.1%), followed by KRAS (45.2%) and PIK3CA (6.7%). Patients with KRAS mutant tumors and wild-type TP53 genotype were found to have no personal history of gastrointestinal cancer (*p* = 0.02, *p* = 0.007). KRAS mutations in exon 3 were associated with the female gender (*p* = 0.02) and the absence of lymph node invasion (*p* = 0.02). PIK3CA mutations were linked to the absence of lymph node invasion (*p* = 0.006). TP53 mutations were associated with KRAS mutations in exon 2 (*p* = 0.006), ulcerated histopathologic type (*p* = 0.04), and G2 differentiation (*p* = 0.01). It provides novel insights into genetic variations specific to the population from Northeastern Romania, which has been underrepresented in previous studies within Eastern Europe. Furthermore, our findings enable the development of genetic profiles in a developing country with limited access to specialized genetic tests and facilitate comparisons with other populations.

## 1. Introduction

Colorectal cancer (CRC) is a highly prevalent cancer worldwide and the third most common neoplasia in Europe [1]. It predominantly affects older adults, with a median age at diagnosis of around 70 years [2]. Incidence and mortality rates of CRC vary significantly across regions and countries, often linked to socioeconomic factors (e.g., higher rates in Europe and North America) [1,3,4]. Furthermore, there are variations in CRC incidence among European countries, with Central and Eastern Europe reporting the highest numbers. In terms of mortality, Romania holds a leading position in Europe, likely due to lifestyle factors, inadequate screening programs, and disparities in therapeutic approaches [5]. CRC arises from glandular epithelial cells in the colon or rectum due to aberrant cell proliferation, following a multistage carcinogenic process characterized by the gradual accumulation of genetic and epigenetic alterations. These alterations primarily result in the hyperactivation of two major signaling pathways: Ras-Raf-MEK-ERK and PI3K/AKT/mTOR [6]. Numerous studies have consistently found high frequencies of RAS, BRAF, and PIK3CA mutations in CRC, with prevalence ranging from 30% to 50%, 10% to 15%, and 10% to 20%, respectively. KRAS variants are present in approximately 40% of cases [7], while NRAS mutations occur in 3–5% of cases [8]. Although RAS gene mutations have been associated with specific clinical, pathological, and molecular characteristics, the exact effects are not yet clearly defined. In clinical practice, KRAS and NRAS mutations serve as biomarkers of resistance to anti-epidermal growth factor receptor (EGFR) monoclonal antibodies in the metastatic setting. They are also linked to unfavorable prognosis in most studies [9]. BRAF mutations have a prevalence of 10–12% (more frequent in females, individuals over 70 years old, and those with poorly differentiated tumors located in the right colon, which exhibit a higher propensity for peritoneal metastasis) and are associated with poor prognosis [10]. In addition to RAS and BRAF genes, which are recommended for testing according to the European Society of Medical Oncology (ESMO) guidelines [10], other biomarkers such as PIK3CA and TP53 gene mutations have been investigated for their clinical utility. PIK3CA mutations are identified in 10–15% of metastatic CRC (mCRC) patients, and a recent meta-analysis suggested their association with proximal tumor location, mucinous histological subtype, and co-occurrence with KRAS mutations. However, their predictive and prognostic significance remains unclear [11].

TP53 mutations play a role in the late-stage tumorigenesis of sporadic CRC, with a prevalence of approximately 55–60% [12]. They are associated with distal colon location, advanced tumor stage, lymph node involvement, metastasis (TNM stage), and a poor prognosis [13]. However, there is limited research on their clinical and pathological associations in stage IV CRC [14]. The frequency of KRAS, BRAF, PIK3CA, and TP53 mutations has primarily been studied in Western countries. In Eastern Europe and specifically Romania, there are limited data on the prevalence and characteristics of these mutations, as well as their clinical, histopathological, and molecular correlations. Among these studies is the research conducted by Negru et al., which focused on the prevalence and characteristics of KRAS mutations in exons 2, 3, and 4, NRAS mutations in exons 2, 3, and 4, and BRAF mutations in exon 15 in the metastatic stage. The study included 161 patients from Romania. However, this study did not explore correlations with clinical, histopathological, or molecular parameters, nor did it include data on PIK3CA and TP53 mutations [15].

The objective of this study is to investigate the frequency, distribution, and characteristics of KRAS, NRAS, BRAF, PIK3CA, and TP53 gene mutations in a population from Romania with metastatic colorectal cancer. Additionally, we aim to analyze the clinical, histopathological, and molecular correlations in order to establish a comprehensive genetic profile. This research aims to facilitate the identification of distinct patient subgroups based on their genetic profiles, taking into account the limited accessibility of genetic testing in individuals residing in developing countries.

## 2. Results

In this study, 104 samples were analyzed to describe the frequency and distribution of mutations and their correlations with clinicopathological features. In the study group, male patients were more numerous than female patients (62.5% vs. 37.5%). The median age in the study group was 64 years, and the majority of patients were 65 years or older (53.8%). A small percentage of patients had a family history of gastrointestinal cancers (5.8%) or a personal history of cancer (5.8%). The primary tumor’s sideness was categorized according to its location: right-sided tumors were considered if they occurred in the ascending colon and two-thirds of the transverse colon from right to left, while left-sided tumors were located in one-third of the transverse colon from left to right, and in the descending and sigmoid colon.

The primary tumor was located in a high percentage in the left colon (78.8%). In terms of the TNM stage, most tumors were T3 (51.9%) and T4 (41.3%), with N1 (45.2%) and N2 (41.3%) lymph node invasion, and M1a metastases (62.5%). Liver metastases were the most frequent (59.6%), followed by peritoneal metastases: (34.6%). In our study we introduced the category of subjects with other sites of metastases to classify all of the patients with secondary lesions which were less frequent like: adrenal gland, bones and brain. Data on clinical and pathological features of the studied group are given in Table 1.

### 2.1. Frequency and Distribution of Mutations

Among the analyzed genes, TP53 exhibited the highest mutation frequency, followed by KRAS and PIK3CA. KRAS mutations were detected in 45.2% of tumor samples, primarily in exon 2 (40.4%) and exon 3 (2.9%). NRAS mutations were observed in 4.8% of cases, with the majority occurring in exon 2 (1%) and exon 3 (3.8%). BRAF mutations were identified in 2.9% of individuals, exclusively in exon 15. PIK3CA mutations were found in 6.7% of patients, with alterations in exon 9 (2.8%) and exon 20 (3.8%). TP53 mutations were observed in 73.1% of cases, with the highest frequency in exon 5 (22.1%), followed by exons 7 (18.2%) and 8 (17.3%). Two new variants have been described in our study that have not been previously identified in international databases (ClinVar, COSMIC and VarSome). For detailed information on mutation frequencies and distributions, please refer to Table 2.

### 2.2. The Clinicopathological Characteristics of Mutations

#### 2.2.1. Coexistence of Mutations

**Figure 1:**
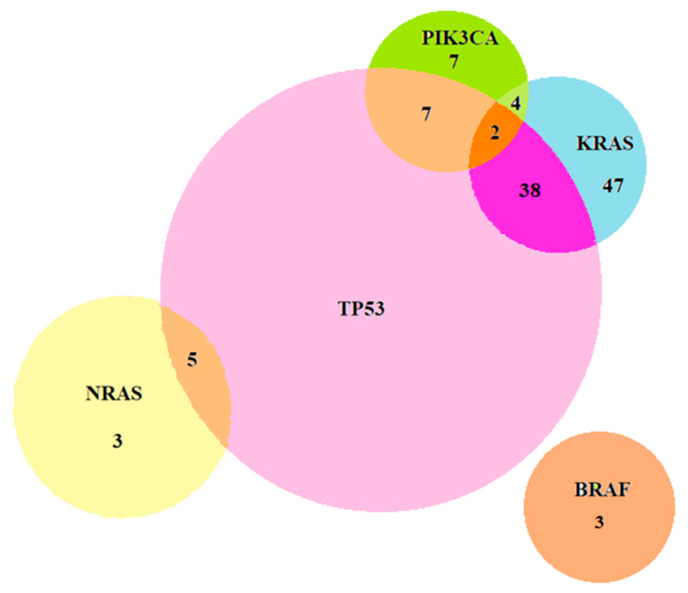
Concomitant mutations.

Among the patients, 11.5% had no mutations, while 88.5% presented with at least one mutation. Of all patients, 35.6% had a single mutation, 48.1% had two coexisting mutations, and 4.8% had three concurrent mutations. The most common double mutations were KRAS exon 2/TP53 (31.7%) and KRAS exon 2/PIK3CA (3.8%). Triple mutations were identified in 1.9% of patients, involving various combinations of KRAS, PIK3CA, and TP53. KRAS mutations were mutually exclusive with NRAS and BRAF, while NRAS mutations were mutually exclusive with BRAF and PIK3CA. No concurrent mutations were observed in four or five of the genes. The distribution of double and triple mutations across the genes can be seen in Figure 1.

#### 2.2.2. Clinical, Pathological and Molecular Correlations

These correlations are detailed in Table 3 and Table 4. Patients with wild-type KRAS had a higher prevalence of a personal history of gastrointestinal cancer compared to patients with KRAS mutations (5.8% vs. 0%, *p* = 0.02). BRAF mutations were more frequently identified in females than in males (2.9% vs. 0%, *p* = 0.02). Tumors located in the right colon exhibited a higher frequency of BRAF mutations than those in the left colon (1.9% vs. 1%, *p* = 0.05). Lung lesions were more common in patients with BRAF mutations compared to those without mutations (1.9% vs. 1%, *p* = 0.02), while liver metastasis was less prevalent in patients with BRAF mutations (0% vs. 3.8%, *p* = 0.03). PIK3CA mutations were inversely associated with lymph node invasion (*p* = 0.006). The presence of TP53 mutation was correlated with an increased occurrence of ulcerated histopathologic type and moderate differentiation in tumors (62.5% vs. 18.3%, *p* = 0.04; 63.5% vs. 19.2%, *p* = 0.01). 

We further investigated the associations between different subtypes of KRAS, NRAS, BRAF, PIK3CA, and TP53 mutations and the clinicopathological and molecular characteristics of the patients. Among these subtypes, significant statistical correlations were observed only in KRAS mutations. The results are presented in Table 5 and Table 6. Patients with TP53 mutations had a higher frequency of KRAS mutations in exon 2 compared to patients without TP53 mutations (34.6% vs. 4.8%, *p* = 0.006). KRAS mutations in exon 3 were more frequently associated with the female sex than the male sex (2.9% vs. 0%, *p* = 0.02), as well as with the absence of lymph node invasion (*p* = 0.02). Tumors with wild-type TP53 gene more often had no personal history of gastrointestinal cancer compared to those with TP53 mutations (93.3% vs. 4.8%, *p* = 0.007).

## 3. Discussion

### 3.1. Frequency and Distribution of Mutations

The frequency of KRAS mutations in metastatic colorectal cancer (mCRC) exhibits global variation. It is approximately 38% in Caucasians, close to 40% in Asians, and only 21% in Africans [16,17]. In our research, out of 104 primary tumor samples of mCRC, 45.2% contained KRAS mutations. These findings are consistent with the varying frequencies observed globally and are in line with previous European studies [9,16,17].

In a pooled analysis of data from 3196 mCRC patients across 36 nations, varying estimates of the prevalence and location of KRAS mutations were reported. KRAS mutations were found in approximately 42.6% in exon 2, 2.9–4.9% in exon 3, and 5.0–7.6% in exon 4 [17]. Consistent with other studies, the majority of KRAS mutations (about 90%) were observed in exon 2, with the most common alterations occurring in codon 12 [18]. Our findings align with this pattern, with 40.4% of mutations in exon 2, predominantly in codon 12. A comparison with an Italian study showed slight differences in the distribution of specific mutations in codon 12, such as G12D, G12V, and G12C [19]. In our study, the prevalence of the KRAS G12C mutation was 7.6%, indicating a higher frequency compared to other reports [20]. 

Mutations in exon 3 were relatively rare, accounting for approximately 3% of all mutations observed in our study. The mutations A59T, Q61H, and Q61R were equally represented, each accounting for 1% of cases. Consistent with our findings, a previous study has also reported codon 61 as the most frequently affected site in exon 3 [19].

The worldwide incidence of NRAS mutations in mCRC patients ranges between 5% and 8% [21]. Some studies suggest that NRAS mutations may be more common in certain demographic groups, such as African Americans, compared to Caucasians [22]. Like KRAS, NRAS mutations predominantly occur in exon 2 (codons 12 and 13) and exon 3 (codon 61). We observed an incidence of 4.8% for NRAS mutations, which aligns with the reported frequency of 4–5% in the literature [23]. The frequency of NRAS exon 3 mutations (3.8%) was higher than that of NRAS exon 2 mutations (1%). Comparing our study with a Danish study, we found similar distributions of NRAS mutations. However, some differences were observed in exon 2 mutations, with G13R reported in our study (1%) and G12D reported as the prevalent NRAS mutation in the Danish population (1.3%) [17].

BRAF-activating mutations are present in 5–15% of cases in patients with mCRC and are often mutually exclusive with KRAS mutations [24]. These mutations are associated with a poor prognosis. However, our study focused only on BRAF V600E mutations, and we identified three patients (2.9%) with such mutations in exon 15. This frequency is lower than what has been reported in Western countries [25,26]. Further research with a larger study group would be beneficial to understand the prevalence of non-V600E BRAF mutations.

Mutations in the PIK3 pathway are found in around 30% of colorectal cancers. In our study, we detected PIK3CA mutations at a lower frequency of 6.7%. Most PIK3CA mutations are located in exon 9 and exon 20, which aligns with our results showing alterations in exon 9 (E545K) and exon 20 (H1047R) [27].

A mutant form of the TP53 gene was detected in 73.1% of our patients, which is higher compared to reports from other populations [28]. However, our results are similar to those observed in an Austrian population. It is worth noting that the Austrian study focused on rectal cancer patients, while our study included primary tumors in multiple regions of the colorectum [29]. 

TP53 mutations were most commonly identified in exon 5 (22.1%) and exon 7 (18.2%) in our study, which is consistent with findings from other publications [28]. In our study, we also observed relatively frequent mutations in exon 10 (7.6%), whereas other studies either mention a lower frequency or do not mention mutations in this exon at all [28]. Regarding the two variants identified for the first time in our population, both were substitutions, and the absence of previous data does not allow us to assess their pathogenicity.

### 3.2. Coexistence of Mutations

Approximately half of the patients had a single mutation. Around 48.1% of tumors had variants in two genes, and 4.8% had variants in three genes. These proportions were lower compared to a Swiss retrospective study, which reported 34% of cases with two mutations and 14% with three mutations [30]. The most common double mutations in our study were KRAS exon 2/TP53 mutations in 31.7% of patients and KRAS exon 2/PIK3CA mutations in 3.8% of patients. As described in other studies, PIK3CA mutations tend to occur together with other genes, most commonly with KRAS [9,31]. Triple mutations were rare in our cohort and in other publications [30]. The co-occurrence of mutations is important when assessing mCRC development, as it may influence treatment decisions. For example, double mutations in RAS and TP53 have clinical implications, as they are associated with worse survival and recurrence compared to mutations in only one or none of these genes, as observed in a retrospective study [32]. However, no prospective studies have evaluated the predictive and prognostic role of concomitant mutations yet [33].

### 3.3. Clinical, Pathological, and Molecular Correlations

KRAS wild-type was associated with a personal history of gastrointestinal cancer. However, this association was not found by other authors and can be attributed to the heterogeneity of our study group. The group without KRAS mutations had slightly more smokers and older patients, which could suggest longer exposure to carcinogens. Another possibility is the higher prevalence of TP53 mutations among patients without KRAS mutations (42/73.6%), as TP53 mutations play a crucial role in preventing cancer development.

We did not find any significant correlations between NRAS mutations and clinicopathological characteristics (Table 3 and Table 4). The same results were obtained in larger European studies [34,35].

BRAF mutant tumors are known to be associated with poor prognosis. Similar to previous studies, we found that BRAF mutations were more frequent in females [36]. Regarding the primary tumor location, our study and other publications have shown that right-sided tumors had a higher rate of BRAF mutations [25]. However, due to the small number of BRAF positive tumors in our study, no meaningful correlations could be identified. 

Our data demonstrated an association between PIK3CA mutations and the absence of lymph node involvement. Similar results were reported in a study of 112 Indian CRC patients [4], while other studies did not find this correlation [37]. However, the association of PIK3CA mutations with clinicopathological features may depend on specific molecular contexts [38].

Moderate differentiation (G2) was associated with tumors carrying TP53 mutations compared to those without TP53 mutations. An analysis of TP53 mutations in a large cohort of CRC patients also revealed a high prevalence of moderately differentiated tumors (605/61.7%) [28]. 

We also found that patients with TP53 mutation had a higher frequency of KRAS mutations in exon 2 compared to patients without TP53 mutation (*p* = 0.006). Similar findings were reported in another study conducted in Romania [39]. This could be attributed to genetic processes that occur early in the development of colorectal cancer.

The rate of KRAS exon 3 mutations was higher in female patients. Since this specific RAS mutation is rare, there are limited data available regarding its association with clinicopathological features. However, this characteristic could be significant in identifying a new subgroup of mCRC patients.

This study provides an overview of RAS, BRAF, PIK3CA, and TP53 mutations in terms of their frequency, distribution, coexistence, and their correlations with clinicopathological and molecular factors in a Romanian population. The observed significant differences compared to other populations could have multiple explanations, including biological factors specific to this population and potential exposure to certain carcinogens. However, it is important to note that our study has methodological limitations such as its retrospective nature and the absence of a rigorously selected study population, which may introduce bias and limit the accuracy of the findings. Because of the small sample size in our study our results need to be carefully regarded and interpreted. Whole genome sequencing can certainly provide a more comprehensive way to characterize the genetic profile of patients in Romania. Unfortunately, due to budget constraints we were not able to perform such analysis. However, these preliminary results gave us support to apply for funding, so we can repeat the experiment with a higher sample size and WGS, and have a much clearer understanding of the genetic profile of mCRC patients and its association to clinicopathological features. Nevertheless, this study represents the first information on the genetic landscape of RAS, BRAF, PIK3CA, and TP53 mutations in the Romanian population with mCRC and offers initial insights into the differences compared to other populations in Europe. The identified correlations can contribute to a better understanding of genetic abnormalities in mCRC and shape molecular profiles that could be used in the future for choosing new evolving treatments, which are now in preclinical or phase I studies. This knowledge may also shed light on the heterogeneity of colorectal cancer and help elucidate the factors influencing tumor behavior and response to therapy.

## 4. Materials and Methods

### 4.1. Study Design

We conducted a retrospective analysis of 104 patients diagnosed with metastatic colorectal cancer (mCRC) who underwent either biopsies or surgical treatment at the Regional Institute of Oncology (RIO), Iași between April 2017 and December 2019. All 104 patients were diagnosed with colon adenocarcinoma. Although all of the subjects were born in the Northeastern part of Romania, no genetic ancestry analysis was performed on them.

### 4.2. Subjects and Data Collection

To be eligible for inclusion in the analysis, patients had to meet the following criteria simultaneously: be over 18 years of age, have a histopathology report confirming colorectal carcinoma, and have a stage IV disease diagnosis certified through imaging and/or pathology results. Patients who had received systemic treatment in the adjuvant or metastatic settings were excluded from the study. The research was conducted in accordance with the principles of the Declaration of Helsinki and was approved by the Ethics Committee of the Regional Institute of Oncology, Iași. Since the study was retrospective in nature, and all patient data were anonymized and reported in aggregate form, specific patient consent was not required.

### 4.3. Methods

The Department of Molecular Biology performed mutation screening on all samples obtained from paraffin-embedded (FFPE) tumor tissue collected during colonoscopy or surgical resection. Five 10 μm thick sections of macrodissected FFPE were used to extract DNA. These sections had to contain at least 50% tumor epithelium, as determined by an experienced pathologist specialized in digestive tract tumors. Next-generation sequencing (NGS) was performed on all 104 cases using the TruSight^®^ Tumor 15 kit for the Illumina platform, which allowed for a comprehensive analysis of 15 genes. Specifically, KRAS exons 2 (partial), 3 (partial), 4; NRAS exons 2 (partial), 3 (partial), 4; BRAF exon 15 (partial); PIK3CA exons 9 and 20; and TP53 exons were evaluated in every sample. It is important to underline that the TP53 gene was fully sequenced. All testing was conducted following the manufacturer’s instructions.

Tumor DNA was isolated from unstained FFPE sections, and areas for macrodissection were identified on the hematoxylin-eosin slide in the pathology department. Only slides with tumor content exceeding 20% were included in the study. 

Paraffin removal was carried out using mineral oil, and DNA isolation was performed using the ReliaPrep FFPE gDNA Miniprep System (Promega), following the manufacturer’s instructions. 

DNA quality (260/280, 260/230) was evaluated using the Nanodrop 2000 (Thermo Scientific, Hong Kong, China), and concentration was determined more accurately using the Qubit dsDNA HS assay kit (Thermo Fisher Scientific, Waltham, MA, USA). Additionally, the extracted DNA was amplified with different sets of primers according to the “BIOMED-2 protocols with DNA extracted from paraffin-embedded tissue biopsies and development of control gene primer set”. Only samples with proper fragmentation (>400 pb) were used for library amplification.

For the NGS TruSight Tumor 15 panel (Illumina, San Diego, CA, USA), libraries were prepared using 20 ng of genomic DNA input, following the manufacturer’s protocol. This kit targets 15 high-risk genes associated with cancer and allows for complete sequencing of the coding region on TP53. The enriched libraries were quantified using the Qubit dsDNA HS assay kit. Individual libraries with a molarity of 2 nmol/L were pooled in batches of eight samples. Up to 8 pmol/L of the pooled library was subjected to cluster generation on the MiSeq flow cell. Paired-end sequencing was performed using the MiSeq Reagent Kit version 3, 600 cycles. Demultiplexing of data and generation of FASTQ sequencing file were carried out in BaseSpace Sequence Hub (Illumina). Alignment to the human reference sequence hg19, variant calling, and annotation were performed using the Illumina Variant Calling Assessment Tool. Variants with a quality score below Q20 were excluded. Only variants with a cumulative depth of 1000 or an average depth of 500x per pool, and a variant frequency of ≥3%, were marked as PASS in the variant call file. Loci with variant frequencies <10%, variant quality < Q30, or depth < 500 for a mutation or reference were filtered out, as well as variants with a frequency >1% in all populations. Nonsynonymous and known or likely pathogenic variants, were manually curated. Only variants annotated in canonical transcripts, predicted as pathogenic/likely pathogenic in the COSMIC database, and confirmed in ClinVar, were retained. Lastly, sequence variants were evaluated for their pathogenicity based on previous literature and databases (COSMIC, ClinVar, OncoKB, Varsome). Mutations were classified as pathogenic, likely pathogenic, variant of unknown significance (VUS), likely benign, or benign. 

To assess the clinical significance of each variant, a comprehensive search was conducted in the ClinVar and COSMIC databases. Subsequently, these variants were documented in Table 2 and categorized symbolically as either pathogenic or new variants.

### 4.4. Statistical Analysis

Statistical analysis was performed using SPSS v25.0 (SPSS, Inc, Chicago, IL, USA). Group comparisons were made using a Chi-square test, while quantitative and ordered variables were compared using the Mann–Whitney U test. For comparisons among three or more groups, a one-way ANOVA analysis was used. A two-sided *p* value ≤ 0.05 was considered statistically significant.

## 5. Conclusions

This single-center retrospective analysis provides the first information on RAS, BRAF, PIK3CA, and TP53 mutation prevalence, distribution, coexistence, and clinicopathological correlations in Romanian mCRC patients. Prospective studies encompassing a larger number of patients are required to thoroughly analyze the clinical, pathological, and molecular aspects of individuals with mCRC, which will make it possible to identify new molecular subtypes. The genetic information presented in this manuscript serves as a valuable asset for the analysis of mCRC in understudied populations.

## Figures and Tables

**Table 1:** Characteristics of the study population.

No. of Patients (%)	104 (100%)	
Median age (years)	64	
Sex	Male	65 (62.5)
	Female	39 (37.5)
Age	<65 years	48 (46.2)
	≥65 years	56 (53.8)
Family history of GI cancer	No	98 (94.2)
	Yes	6 (5.8)
Personal history of cancer	No	98 (94.2)
	Yes	6 (5.8)
Smoking status	Non-smokers	98 (94.2)
	Smokers	6 (5.8)
Tumor location	Left colon	82 (78.8)
	Right colon	22 (21.1)
T stage	T1	1 (1)
	T2	6 (5.8)
	T3	54 (51.9)
	T4	43 (41.3)
N stage	N0	14 (13.5)
	N1	47 (45.2)
	N2	43 (41.3)
M stage	M1a	65 (62.5)
	M1b	16 (15.4)
	M1c	23 (22.1)
Location of metastases	Liver	62 (59.6)
	Peritoneal	36 (34.6)
	Lung	18 (17.4)
	Other sites	9 (8.7)
Histopathological type	Ulcerated	84 (80.8)
	Mucinous	16 (15.4)
	Signet ring cell	4 (3.8)
Grading	G1	8 (7.7)
	G2	86 (82.7)
	G3	10 (9.6)

**Table 2:** Frequency and distribution of mutations.

Gene	Reference Sequence	Nucleotide Change	Amino Acid Change	Variant Frequency	Cases (%)
KRAS exon 2					42 (40.4%)
	NM_004985.5	c.35G > A ^‡^	p.Gly12Asp	(0.257–0.384)	13
	NM_004985.5	c.34G > T ^‡^	p.Gly12Cys	(0.043–0.339)	8
	NM_004985.5	c.35G > T ^‡^	p.Gly12Val	(0.118–0.587)	8
	NM_004985.5	c.34G > A ^‡^	p.Gly12Ser	(0.212–0.669)	5
	NM_004985.5	c.38G > A ^‡^	p.Gly13Asp	(0.208–0.291)	5
	NM_004985.5	c.35G > C ^‡^	p.Gly12Ala	(0.469–0.541)	2
	NM_004985.5	c.37G > T ^‡^	p.Gly13Cys	0.099	1
KRAS exon 3					3 (2.9%)
	NM_033360.4	c.175G > A ^‡^	p.Ala59Thr	0.318	1
	NM_004985.5	c.183A > T ^‡^	p.Gln61His	0.090	1
	NM_004985.5	c.182A > G ^‡^	p.Gln61Arg	0.558	1
KRAS exon 4					2 (1.9%)
	NM_033360.4	c.351A > T ^‡^	p.Lys117Asn	0.410	1
	NM_004985.5	c.436G > A ^‡^	p.Ala146Thr	0.793	1
NRAS exon 2					1 (1%)
	NM_002524.5	c.37G > C ^‡^	p.Gly13Arg	0.529	1
NRAS exon 3					4 (3.8%)
	NM_002524.5	c.181C > A ^‡^	p.Gln61Lys	(0.271–0.489)	3 (2.9%)
	NM_002524.5	c.182A > T ^‡^	p.Gln61Leu	0.538	1 (1%)
BRAF exon 15					3 (2.9%)
	NM_004333.6	c.1799T > A ^‡^	p.Val600Glu	(0.130–0.446)	3
PIK3CA exon 9					3 (2.9%)
	NM_006218.4	c.1633G > A ^‡^	p.Glu545Lys	(0.026–0.259)	3
PIK3CA exon 20					4 (3.8%)
	NM_006218.4	c.3140A > G ^‡^	p.His1047Arg	(0.022–0.216)	4
TP53 exon 9					1 (1%)
	NM_000546.6	c.958A > T ^‡^	p.Lys320Ter	0.371	1 (1%)
TP53 exon 10					8 (7.6%)
	NM_000546.6	c.1009C > T ^‡^	p.Arg337Cys	(0.027–0.032)	6
	NM_000546.6	c.1024C > T ^‡^	p.Arg342Ter	(0.025–0.481)	2
TP53 exon 4					5 (4.8%)
	NM_000546.6	c.158G > A ^‡^	p.Trp53Ter	(0.460–0.688)	2
	NM_000546.6	338T > A ^‡^	p.Val272Glu	0.333	2
	NM_000546.6	c.151G > T ^‡^	p.Glu51Ter	0.205	1
TP53 exon 5					23 (22.1%)
	NM_000546.6	c.524G > A ^‡^	p.Arg175His	(0.066–0.847)	11
	NM_000546.6	c.527G > A ^‡^	p.Cys176Tyr	(0.026–0.256)	2
	NM_000546.6	c.535C > T ^‡^	p.His179Tyr	(0.089–0.659)	2
	NM_000546.6	c.395A > G ^‡^	p.Lys132Arg	0.531	1
	NM_000546.6	c.473G > A ^‡^	p.Arg158His	(0.182–0.456)	2
	NM_000546.6	c.379T > C ^‡^	p.Ser127Pro	0.233	1
	NM_000546.6	c.380C > T ^‡^	p.Ser127Phe	0.248	1
	NM_000546.6	814G > T ^‡^	p.Val272Leu	0.034	1
	NM_000546.6	376T > G ^¥^	p.Tyr126Asp	0.351	1
	NM_000546.6	c.377A > G ^‡^	p.Tyr126Cys	0.452	1
TP53 exon 6					4 (3.8%)
	NM_000546.6	c.569C > T ^‡^	p.Pro190Leu	0.600	1
	NM_000546.6	c.586C > T ^‡^	p.Arg196Ter	0.339	1
	NM_000546.6	637C > T ^‡^	p.Arg213Ter	0.263	1
	NM_000546.6	644G > A ^‡^	p.Ser215Asn	0.328	1
TP53 exon 7					19 (18.2%)
	NM_000546.6	c.742C > T ^‡^	p.Arg248Trp	(0.052–0.334)	8
	NM_000546.6	c.743G > A ^‡^	p.Arg248Gln	(0.038–0.729)	6
	NM_000546.6	c.772G > A ^‡^	p.Glu258Lys	0.531	1
	NM_000546.6	c.730G > T ^‡^	p.Gly244Cys	0.049	1
	NM_000546.6	c.733G > A ^‡^	p.Gly245Ser	0.465	1
	NM_000546.6	c.723delC ^‡^	p.Cys242AlafsTer5	0.628	1
	NM_000546.6	c.747delG ^‡^	p.Arg249SerfsTer96	0.512	1
TP53 exon 8					18 (17.3%)
	NM_000546.6	c.844C > T ^‡^	p.Arg282Trp	(0.089–0.609)	5
	NM_000546.6	c.817C > T ^‡^	p.Arg273Cys	(0.173–0.736)	4
	NM_000546.6	c.814G > A ^‡^	p.Val272Met	(0.096–0.647)	3
	NM_000546.6	c.818G > A ^‡^	p.Arg273His	(0.137–0.324)	2
	NM_000546.6	c.880G > T ^‡^	p.Glu294Ter	0.180	1
	NM_000546.6	c.833C > T ^‡^	p.Pro278Leu	0.286	1
	NM_000546.6	c.839G > T ^‡^	p.Arg280Ile	0.447	1
	NM_000546.6	c.821T > G ^‡^	p.Val274Gly	0.499	1

Legend: ^‡^ Pathogenic; ^¥^ New variant.

**Table 3:** Clinical characteristics of patients with KRAS, NRAS, BRAF, PIK3CA and TP53 mutations. ^a^ Chi-square test; ^b^ Mann–Whitney test.

		KRAS Mutation			NRAS Mutation			BRAF Mutation			PIK3CA Mutation			TP53 Mutation		
	Case	No, (n%)	Yes, (n%)	*p* Value	No, (n%)	Yes, (n%)	*p* Value	No, (n%)	Yes, (n%)	*p* Value	No, (n%)	Yes, (n%)	*p* Value	No,	Yes, (n%)	*p* Value
	104, n (%)													**(n%)**		
Sex				0.5 ^a^			0.4 ^a^			**0.02** ^a^			0.76 ^a^			0.81 ^a^
Male	65 (62.5)	34 (32.7)	31 (29.8)		61 (58.6)	4 (3.8)		65 (62.5)	0 (0)		61 (60.6)	4 (3.8)		18 (17.3)	47 (45.2)	
Female	39 (37.5)	23 (22.1)	16 (15.4)		38 (36.5)	1 (1)		36 (34.6)	3 (2.9)		36 (34.6)	3 (2.9)		10 (9.6)	29 (27.9)	
Age				0.19 ^a^			0.77 ^a^			0.1 ^a^			0.85 ^a^			0.39 ^a^
<65	48 (46.2)	23 (22.2)	25 (24)		46 (44.3)	2 (1.9)		48 (46.1)	0 (0)		45 (43.2)	3 (2.9)		11 (10.5)	37 (35.7)	
≥65	56 (53.8)	34(32.7)	22 (21.1)		53 (50.9)	3 (2.9)		53 (50.9)	3 (2.9)		52 (50)	4 (3.8)		17 (16.3)	39 (37.5)	
Tumor location				0.64 ^a^			0.94 ^a^			**0.05** ^a^			0.64 ^a^			
Left colon	82 (78.8)	44 (42.3)	38 (36.5)		78 (75)	4 (3.8)		81 (77.8)	1 (1)		76 (73)	6 (5.8)		20 (19.2)	62 (59.6)	0.26 ^a^
Right colon	22 (21.1)	13 (12.5)	9 (8.7)		21 (20.1)	1 (1)		20 (19.2)	2 (1.9)		21 (20.1)	1 (1)		8 (7.7)	14 (13.5)	
T stage				0.47 ^b^			0.22 ^b^			0.34 ^b^			0.25 ^b^			0.59 ^b^
T1	1 (1)	0 (0)	1 (1)		1 (1)	0 (0)		1 (1)	0 (0)		1 (1)	0 (0)		1 (1)	0 (0)	
T2	6 (5.8)	3 (2.9)	3 (2.9)		5 (4.8)	1 (1)		6 (5.8)	0 (0)		6 (5.8)	0 (0)		2 (1.9)	4 (3.8)	
T3	54 (52)	33 (31.7)	21 (20.3)		51 (49)	3 (2.9)		53 (51)	1 (1)		48 (46.2)	6 (5.8)		14 (13.4)	40 (38.6)	
T4	43 (41.3)	21 (20.1)	22 (21.1)		42 (40.3)	1 (1)		41 (39.3)	2 (1.9)		42 (40.4)	1 (1.9)		11 (10.6)	32 (30.7)	
N stage				0.63 ^b^			0.29 ^b^			0.73 ^b^			**0.006** ^b^			0.53 ^b^
N0	14 (13.5)	9 (8.7)	5 (4.8)		14 (13.5)	0 (0)		13 (12.5)	1 (1)		11 (10.6)	3 (2.9)		4 (3.8)	10 (9.6)	
N1	47 (45.2)	25 (24.1)	22 (21.1)		45 (43.3)	2 (1.9)		47 (45.2)	0 (0)		43 (41.3)	4 (3.8)		14(13.5)	33 (31.7)	
N2	43 (41.3)	23 (22.1)	20 (19.2)		40 (38.4)	3 (2.9)		41 (39.4)	2 (1.9)		43 (41.3)	0 (0)		10 (9.6)	33 (31.7)	
M stage				0.91 ^b^			0.95 ^b^			0.97 ^b^			0.4 ^b^			0.63 ^b^
M1a	65 (62.5)	35 (33.7)	30 (28.9)		62 (59.6)	3 (2.9)		63 (60.6)	2 (1.9)		60 (57.7)	5(4.8)		17 (16.3)	48 (46.2)	
M1b	16 (15.4)	10 (9.6)	6 (5.8)		15 (14.4)	1 (1)		16 (15.4)	0 (0)		14 (13.5)	2 (1.9)		3 (2.9)	13 (12.5)	
M1c	23 (22.1)	12 (11.6)	11(10.5)		22 (21.1)	1 (1)		22 (21.1)	1 (1)		23 (22.1)	0 (0)		8 (7.7)	15 (14.4)	
**Liver**				0.23 ^a^			0.36 ^a^			**0.03** ^a^			0.51 ^a^			0.75 ^a^
metastases																
No	42 (40.4)	26 (25)	16 (15.4)		39 (37.5)	3 (2.9)		39 (37.5)	3 (2.8)		40 (38.5)	2 (1.9)		12 (11.5)	30 (28.9)	
Yes	62 (59.6)	31 (29.8)	31 (29.8)		60 (57.7)	2 (1.9)		62 (59.6)	0 (0)		57 (54.8)	5 (4.8)		16 (15.4)	46 (44.2	
Peritoneal				0.17 ^a^			0.79 ^a^			0.96 ^a^			0.72 ^a^			0.54 ^a^
metastases																
No	68 (65.4)	34 (32.7)	34 (32.7)		65 (62.5)	3 (2.9)		66 (63.5)	2 (1.9)		63 (60.6)	5 (4.8)		17 (16.3)	51 (49.1)	
Yes	36 (34.6)	23 (22.1)	13 (12.5)		34 (32.7)	2 (1.9)		35 (33.6)	1 (1)		34 (32.7)	2 (1.9)		11 (10.6)	25 (24)	
Lung				0.65 ^a^			0.16 ^a^			**0.02** ^a^			0.82 ^a^			0.28 ^a^
metastases																
No	86 (82.6)	48 (46.1)	38 (36.5)		83 (79.7)	3 (2.9)		85 (81.6)	1 (1)		80 (76.9)	6 (5.8)		25 (24)	61 (58.6)	
Yes	18 (17.4)	9 (8.7)	9 (8.7)		16 (15.4)	2 (1.9)		16 (15.4)	2 (1.9)		17 (16.4)	1 (1)		3 (2.9)	15 (14.5)	

**Table 4:** Pathological and molecular characteristics of patients with KRAS, NRAS, BRAF, PIK3CA and TP53 mutations. ^a^ Chi-square test; ^b^ Mann–Whitney test.

		KRAS Mutation			NRAS Mutation			BRAF Mutation			PIK3CA Mutation			TP53 Mutation		
	Case 104, (n%)	No, (n%)	Yes, (n%)	*p* Value	No, (n%)	Yes, (n%)	*p* Value	No, (n%)	Yes, (n%)	*p* Value	No, (n%)	Yes, (n%)	*p* Value	No, (n%)	Yes, (n%)	*p* Value
Pathological type				0.47 ^a^			0.53 ^a^			0.69 ^a^			0.54 ^a^			**0.04** ^a^
Ulcerated	84 (80.8)	47 (45.2)	37 (35.6)		79 (75.7)	5 (4.8)		81 (77.9)	3 (2.9)		79 (76)	5 (4.8)		19 (18.3)	65 (62.5)	
Mucinous	16 (15.4)	7 (6.7)	9 (8.7)		16 (15.4)	0 (0)		16 (15.4)	0 (0)		14 (13.5)	2 (1.9)		6 (5.8)	10 (9.6)	
Signet ring cell	4 (3.8)	3 (2.8)	1 (1)		4 (3.8)	0 (0)		4 (3.8)	0 (0)		4 (3.8)	0 (0)		3 (2.8)	1 (1)	
Grading				0.17 ^b^			0.32 ^b^			0.92 ^b^			0.28 ^b^			**0.01** ^b^
G1	8 (7.7)	2 (1.9)	6 (5.8)		8 (7.6)	0 (0)		8 (7.7)	0 (0)		7 (6.7)	1 (1)		6 (5.8)	2 (1.9)	
G2	86 (82.7)	49 (47.2)	37 (35.5)		82 (78.8)	4 (3.8)		83 (79.8)	3 (2.9)		80 (76.9)	6 (5.8)		20 (19.2)	66 (63.5)	
G3	10 (9.6)	6 (5.8)	4 (3.8)		9 (8.6)	1 (1)		10 (9.6)	0 (0)		10 (9.6)	0 (0)		2 (1.9)	8 (7.7)	
KRAS mutation							**0.03** ^a^			0.11 ^a^			0.51 ^a^			0.87 ^a^
Negative	57 (54.8)				52 (50)	5 (4.8)		54 (52)	3 (2.9)		54 (51.9)	3 (2.9)		15 (14.4)	42 (40.4)	
Positive	47 (45.2)				47 (45.2)	0 (0)		47 (45.2)	0 (0)		43 (41.3)	4 (3.8)		13 (12.5)	34 (32.7)	
BRAF mutation				0.11 ^a^			0.69 ^a^						0.63 ^a^			0.11 ^a^
Negative	101 (97.1)	54 (51.9)	47 (45.2)		96 (92.3)	5 (4.8)					94 (90.4)	7 (6.7)		26 (25)	75 (72.1)	
Positive	3 (2.9)	3 (2.9)	0 (0)		3 (2.9)	0 (0)					3 (2.9)	0 (0)		2 (1.9)	1 (1)	
PIK3CA mutation				0.51 ^a^			0.53 ^a^			0.63 ^a^						0.43 ^a^
Negative	97 (93.3)	54 (51.9)	43 (41.3)		92 (88.5)	5 (4.8)		94 (90.4)	3 (2.9)					27 (26)	70 (67.3)	
Positive	7 (6.7)	3 (2.9)	4 (3.8)		7 (6.7)	0 (0)		7 (6.7)	0 (0)					1 (1)	6 (5.8)	
TP53 mutation				0.87 ^a^			0.72 ^a^			0.11 ^a^			0.43 ^a^			
Negative	28 (26.9)	15 (14.4)	13 (12.5)		27 (26)	1 (1)		26 (25)	2 (1.9)		26 (25)	2 (1.9)				
Positive	76 (73.1)	42 (40.4)	34 (32.7)		72 (69.3)	4 (3.8)		75 (72.1)	1 (1)		73 (70.2)	3 (2.9)				

**Table 5:** Clinical and pathological characteristics of KRAS mutations by exon subtype. ^a^ Chi-square test; ^b^ Mann–Whitney test.

			KRAS Exon 2 Mutation	
	Case 104, (n%)	No, (n%)	Yes, (n%)	*p* Value
Sex				
Male	65(62.5)	38 (58.5)	27 (41.5)	0.56 ^a^
Female	39 (37.5)	25 (64.1)	14 (35.9)	
Age				
<65	48 (46.2)	28 (26.9)	20 (19.2)	0.66 ^a^
≥65	56 (53.8)	35 (33.7)	21 (20.2)	
Family history of GI cancer				
No	98 (60.5)	59 (56.7)	39 (37.5)	0.75 ^a^
Yes	6 (5.8)	4 (3.8)	2 (1.9)	
Personal history of cancer				
No	98 (94.2)	59 (56.7)	39 (37.5)	0.75 ^a^
Yes	6 (5.8)	4 (3.8)	2 (1.9)	
Smoking status				
Non-smokers	98 (94.2)	59 (56.7)	4 (3.8)	0.75 ^a^
Smokers	6 (5.8)	39 (37.5)	2 (1.9)	
Tumor location				
Left colon	82 (78.8)	50 (48.1)	32 (30.8)	0.87 ^a^
Right colon	22 (21.1)	13 (12.5)	9 (8.7)	
T stage				
T1				1 (1)
T2	6 (5.8)	5 (4.8)	1 (1)	
T3	54 (52)	33 (31.7)	21 (20.2)	
T4	43 (41.3)	24 (23.1)	19 (18.3)	
N stage				
N0	14 (13.5)	8 (7.7)	6 (5.8)	0.81 ^b^
N1	47 (45.2)	30 (28.8)	17 (16.3)	
N2	43 (41.3)	25 (24)	18 (17.3)	
M stage				
M1a				65 (62.5)
M1b	16 (15.4)	10 (9.6)	6 (5.8)	
M1c	23 (22.1)	14 (13.5)	9 (8.7)	
Liver metastases				
No	42 (40.4)	28 (26.9)	14 (13.5)	0.29 ^a^
Yes	62 (59.6)	35 (33.7)	27 (26)	
Peritoneal metastases				
No	68 (65.4)	41 (39.4)	27 (26)	0.93 ^a^
Yes	36 (34.6)	22 (21.2)	14 (13.5)	
Lung metastases				
No	86 (82.6)	50 (48.1)	36 (34.6)	0.26 ^a^
Yes	18 (17.4)	13 (12.5)	5 (4.8)	

**Table 6:** Molecular characteristics of KRAS mutations by exon subtype. ^a^ Chi-square test; ^b^ Mann–Whitney test.

			KRAS Exon 2 Mutation	
	Case 104, (n%)	No, (n%)	Yes, (n%)	*p* Value
Histopathological type				0.81 ^a^
Ulcerated	84 (80.8)	50 (48.1)	34 (32.7)	
Mucinous	16 (15.4)	10 (9.6)	6 (5.8)	
Signet ring cell	4 (3.8)	3 (2.9)	1 (1)	
Grading				
G1	8 (7.7)	7 (6.7)	1 (1)	0.12 ^b^
G2	16 (5.4)	51 (49)	35 (33.7)	
G3	4 (3.8)	5 (4.8)	5 (4.8)	
NRAS mutation				
Negative	99 (95.2)	62 (59.6)	37 (35.6)	0.58 ^a^
Positive	5 (4.8)	5 (4.8)	0 (0)	
BRAF				
Negative	101 (97.1)	60 (57.7)	41 (39.4)	0.15 ^a^
Positive	3 (2.9)	3 (2.9)	0 (0)	
PIK3CA mutation				
Negative	97 (93.3)	57 (54.8)	40 (38.5)	0.15 ^a^
Positive	7 (6.7)	6 (5.8)	1 (1)	
TP53 mutation				
Negative	28 (26.9)	23 (22.1)	5 (4.8)	**0.006** ^a^
Positive	76 (73.1)	40 (38.5)	36 (34.6)	

## Data Availability

The data that support the findings of this study are not publicly available but are available from the authors upon reasonable request.
